# A cutaneous nodule revealing Münchmeyer’s disease

**DOI:** 10.1093/omcr/omaf045

**Published:** 2025-05-28

**Authors:** Yasmine Rkiek, Ouiame El Jouari, Mouna Rimani, Meriem Haddad, Salim Gallouj

**Affiliations:** Department of Dermatology, University Hospital Center of Tangier, Tetouan Al Hoceima, Faculty of Medicine and Pharmacy Tangier Abdelmalek Essaadi University, 90100 Tangier, Morocco; Department of Dermatology, University Hospital Center of Tangier, Tetouan Al Hoceima, Faculty of Medicine and Pharmacy Tangier Abdelmalek Essaadi University, 90100 Tangier, Morocco; Department of Pathological Anatomy Hassan, 10020 Rabat, Morocco; Department of Dermatology, University Hospital Center of Tangier, Tetouan Al Hoceima, Faculty of Medicine and Pharmacy Tangier Abdelmalek Essaadi University, 90100 Tangier, Morocco; Department of Dermatology, University Hospital Center of Tangier, Tetouan Al Hoceima, Faculty of Medicine and Pharmacy Tangier Abdelmalek Essaadi University, 90100 Tangier, Morocco

**Keywords:** Münchmeyer’s disease, Fibrodysplasia ossificans progressive, cutaneous nodules, ossification

## Abstract

Münchmeyer’s disease, also known as fibrodysplasia ossificans progressiva, is an extremely rare congenital condition affecting the musculoskeletal system, occurring in approximately 1 in 2 million people. We report the case of a 13-month-old male toddler who presented with dorsal cutaneous nodules. Examination revealed a firm, immobile nodule and congenital hallux valgus with microdactyly. Imaging studies showed ossifications of the subcutaneous tissues, and genetic testing confirmed a mutation in the ACVR1 gene, leading to the diagnosis of Münchmeyer’s disease. This disabling, progressive condition is characterized by extraskeletal ossification and congenital big toe malformations. The precocity of the diagnosis in this case is noteworthy, as Münchmeyer’s disease is often misdiagnosed, leading to significant delays and complications. Awareness of this rare condition is important for dermatologists to avoid missed or delayed diagnoses, as early recognition and appropriate management are crucial.

## Introduction & Objectives

Münchmeyer’s disease, also known as fibrodysplasia ossificans progressiva (FOP) or ‘stone man’s disease’, is an extremely rare and debilitating congenital autosomal dominant disease affecting the musculoskeletal system characterized by heterotopic ossification. It is marked by congenital stigmata of the extremities and extensive soft tissue ossification. The disease is caused by a gain-of-function mutation in the ACVR1 gene, which encodes the activin A receptor type 1, a protein involved in bone morphogenetic protein (BMP) signaling. It occurs in approximately 1 in 2 million people worldwide [1]. We report a case of this rare condition.

## Case report

**Figure 1:**
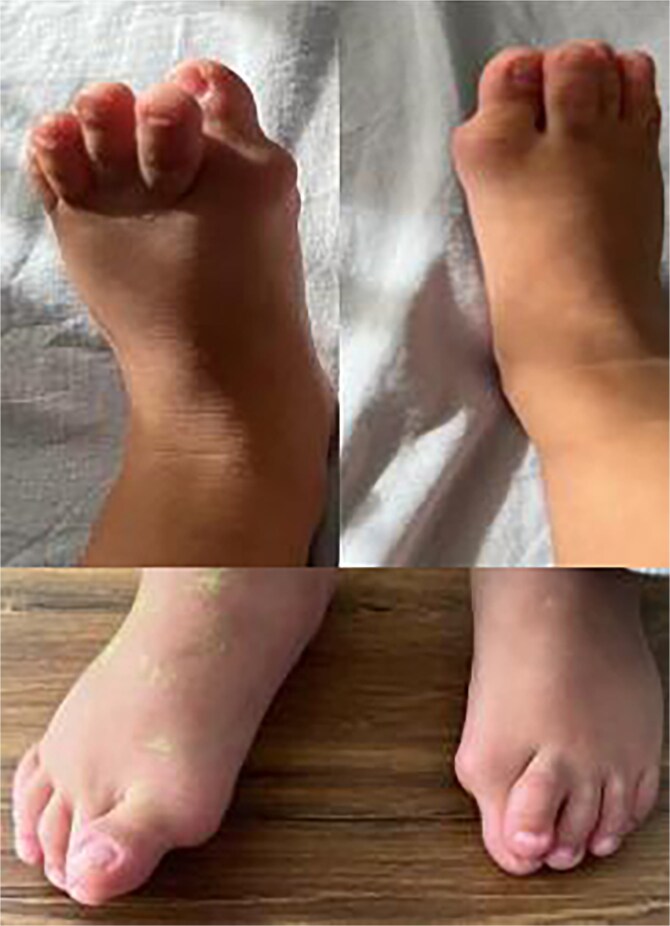
Hallux valgus with microdactyly of the big toes.

**Figure 2:**
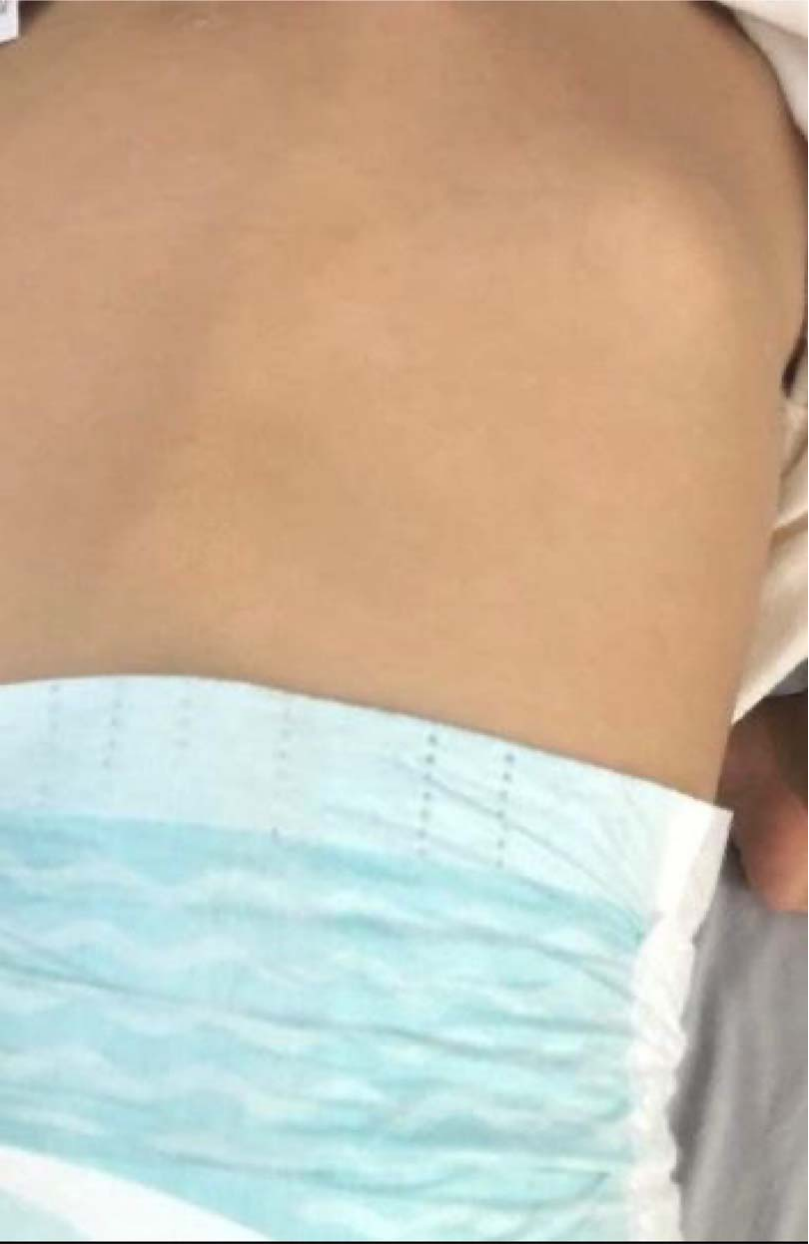
Nodules and tumefaction in the paravertebral region.

**Figure 3:**
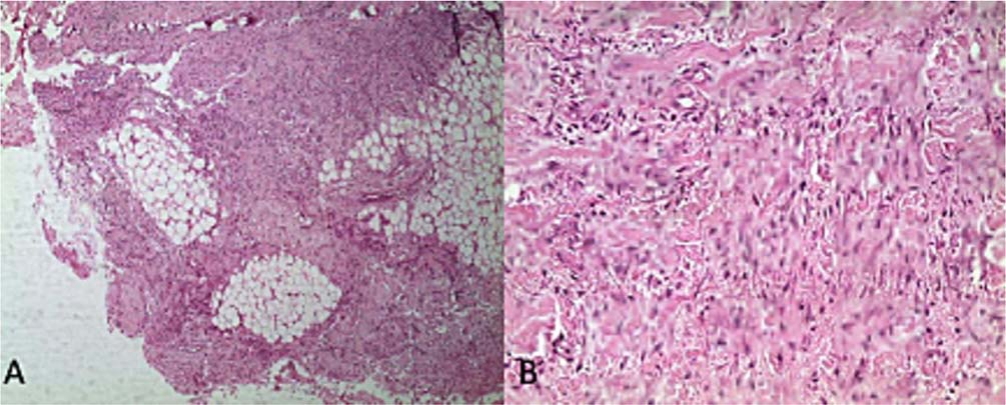
Mesenchymal proliferation of fibroblastic and myofibroblastic spindle cells mixed with mature adipocytes: Histological image (A)*50,(B)*400 stained with hematoxylin and eosin (H&E).

**Figure 4:**
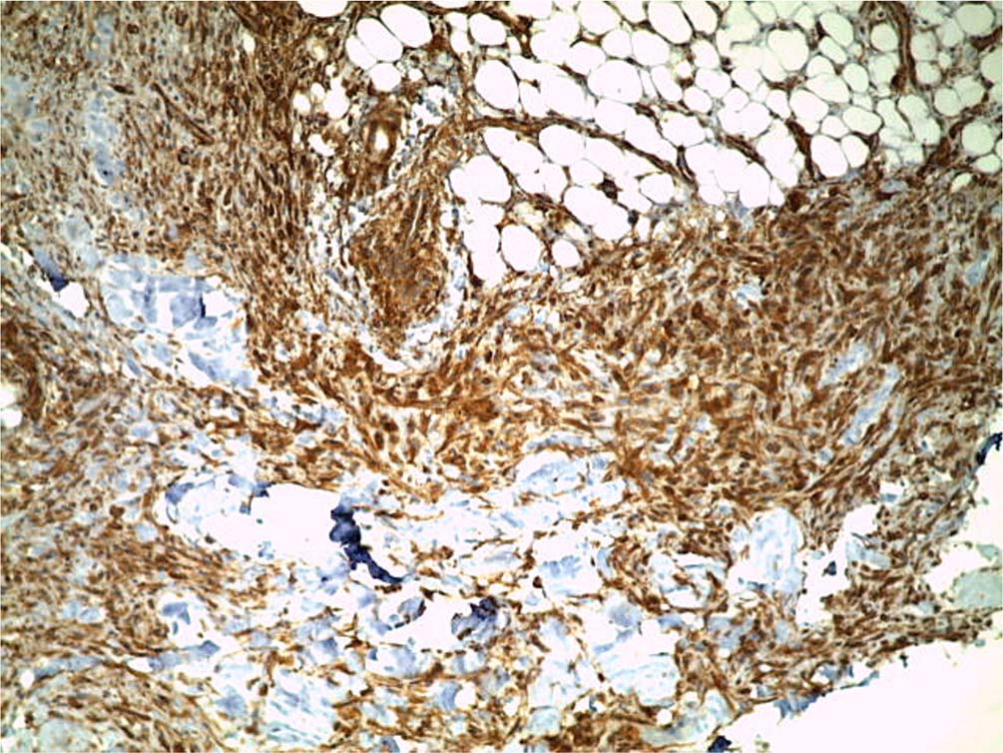
Immunohistochemical study findings with positive labelling of anti-smooth muscle actin antibodies (ASMA).

**Figure 5:**
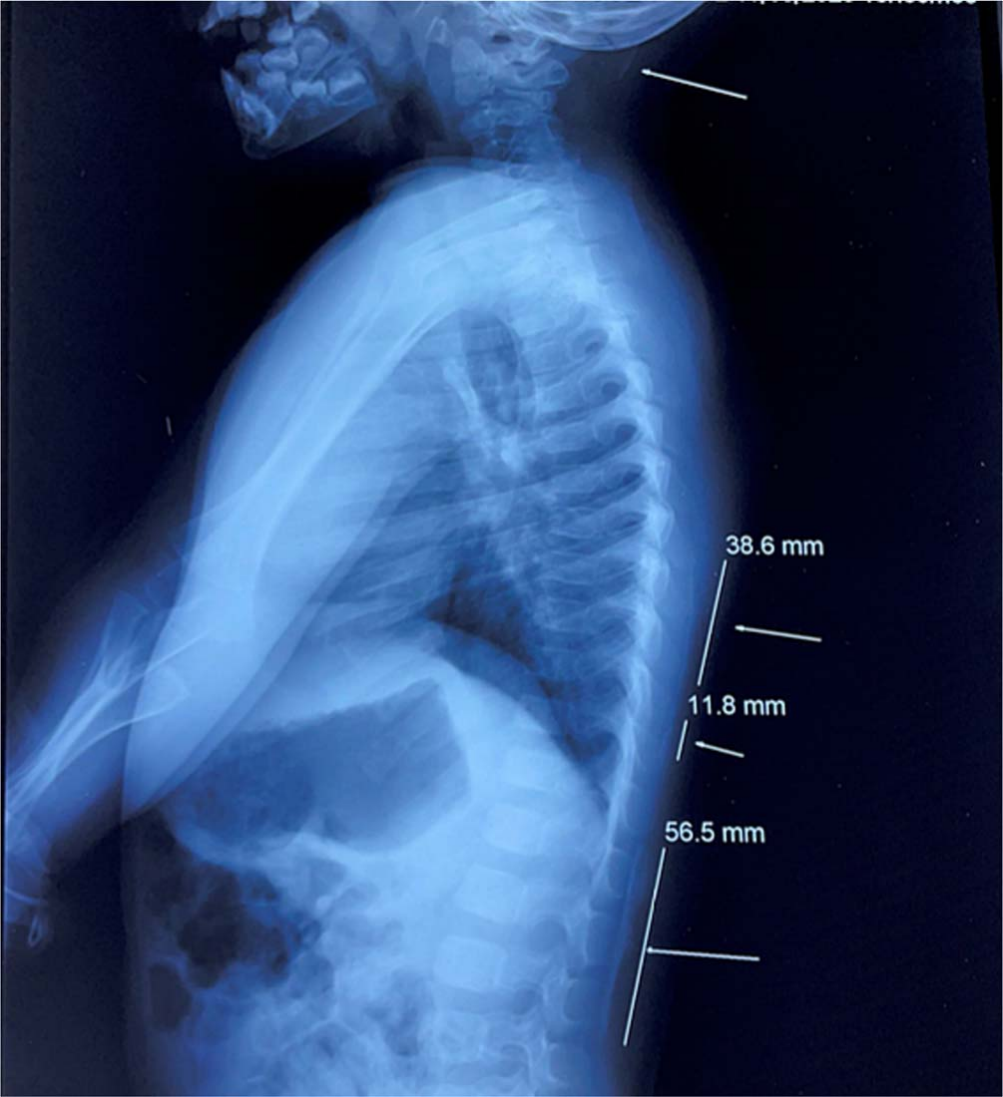
X ray showing ossifications of the subcutaneous soft tissues.

A 13-month-old male toddler, with no notable medical history, presented with a scalp tumefaction since 2 months of age. A CT scan at that time suggested a hematoma, which did not fully regress. Two months prior to presentation, the patient developed a dorsal cutaneous nodule. Examination revealed a firm, immobile, painless nodule with no signs of inflammation. Congenital hallux valgus with microdactyly of the big toes was also noted (Fig. 1). An initial ultrasound suggested an epidermal cyst, but additional nodules and a paravertebral tumefaction soon appeared (Fig. 2). A biopsy showed a mesenchymal proliferation made up of a mixture of mature adipocytic cells, spindle-shaped cells with a fibroblastic and myofibroblastic appearance with hyalinised collagenous (Figs. 3-4) whose morphology and immunohistochemical data are suggestive of conditions like lipofibromatosis, infantile fibrous hamartoma or infantile myofibromatosis. A repeat ultrasound revealed partially calcified tissue masses. X-rays showed ossifications of the subcutaneous soft tissues (Fig. 5) without notable bone abnormalities. The combination of ossifications and hallux valgus malformations was suggestive of fibrodysplasia. Molecular Genetic testing revealed a heterozygous presence of the ACVR1 variant using the Ion AmpliSeq™ Custom DNA Panel (supplied by Thermo Fisher Scientific), a targeted next-generation sequencing (NGS) technology.This variant is classified as pathogenic (Class 5) according to the criteria of the American College of Medical Genetics and Genomics. The ACVR1 gene is notably associated with the OMIM phenotype 135 100 (Fibrodysplasia ossificans progressiva), leading to the diagnosis of Münchmeyer’s disease.

The patient was initiated on symptomatic treatment. He was placed on corticosteroids combined with pamidronic acid. Counseling was provided for the parents, and prevention of trivial trauma was advised.

**Figure 6:**
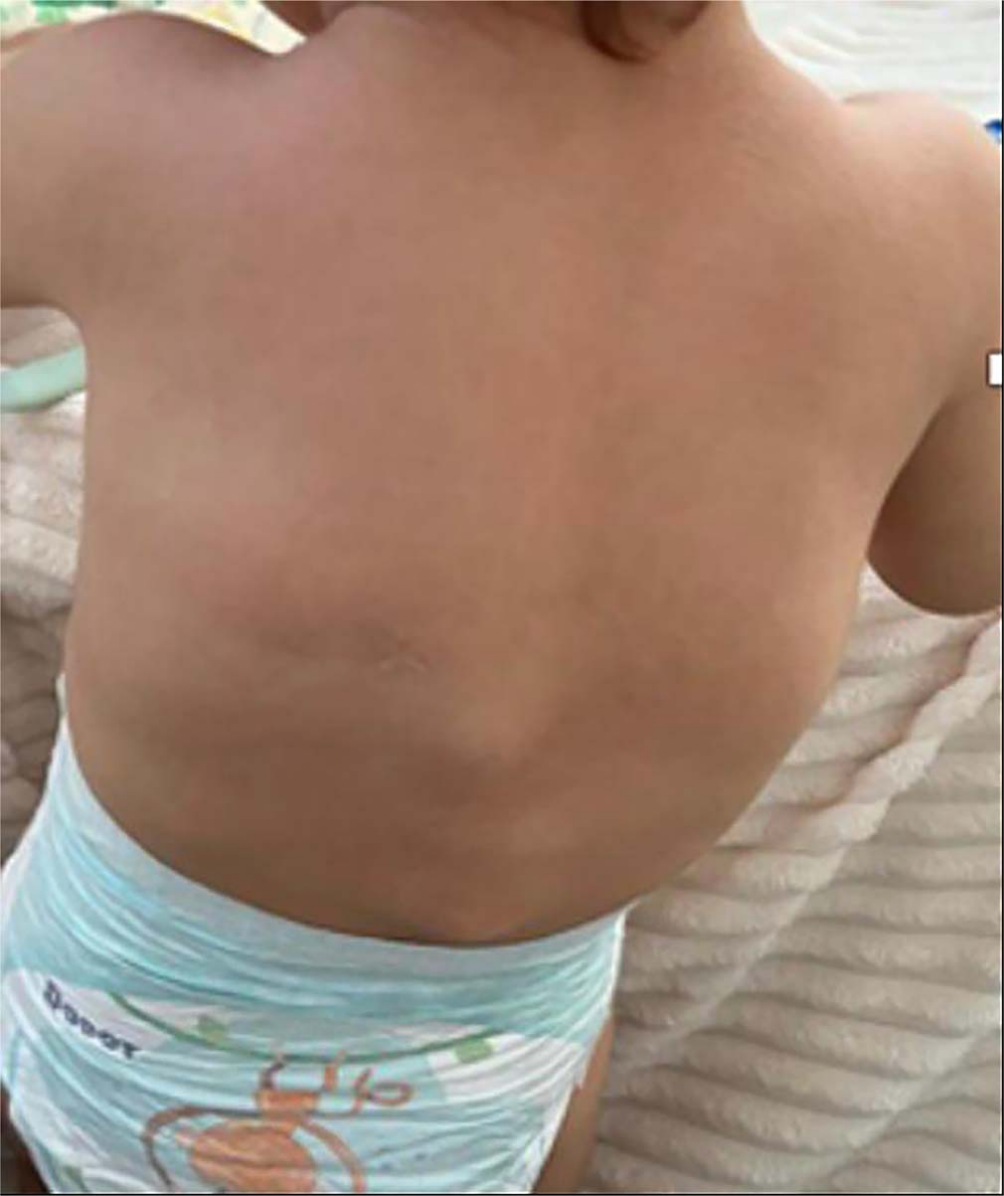
Ectopic ossification lesions in the dorsal region.

The evolution was marked by the involution of nodules at the beginning, but two months later new lesions appeared (Fig. 6). The patient was replaced on corticosteroids, and has just started tofacitinib as a part of a clinical trial.

## Discussion

Münchmeyer’s disease is a disabling, progressive condition characterized by extraskeletal bone formation that gradually encases the original skeleton, along with congenital malformations of the big toes, in particular short and valgus deformed big toes [2]. The reported worldwide prevalence is approximately 1 in 2 million, with no ethnic, racial, sexual or geographic predilection [3]. Patin was the first to describe it in 1962 [1].

The disease begins early, before the age of 3, and aggressively affects the musculoskeletal system.

The initial phase of the disease is often poorly diagnosed due to its rarity and lack of recognition [4].

Ossification is episodic, but the disability is cumulative. In the first decade of life, FOP usually starts with painful swelling in the soft tissues, often triggered by minor injuries or infections, leading to early bone formation. By the second decade, ossification continues to spread, progressively limiting joint mobility and causing joint contractures. This results in increasing disability, with many individuals losing the ability to walk or perform daily tasks. By late adolescence, most patients experience severe immobility, require lifelong assistance with daily activities, and are confined to a wheelchair by their third decade of life [5].

The originality and the particularity of this observation reside not only in the rarity of this disease but also and especially in the precocity of the diagnosis. If we look at the literature, we find that the average age of diagnosis is around 5 years [5], with extremes of 37 years in one case report [6].

According to Kitterman et al., misdiagnosis rates exceed 90%, with 67% of patients undergoing unnecessary invasive procedures, and 68% receiving inappropriate therapies. These interventions often lead to severe consequences, with 49% of patients experiencing permanent loss of mobility due to posttraumatic ossification caused by these procedures [7]. While an accurate diagnosis cannot prevent the lifelong disability associated with FOP, it plays a crucial role in reducing the risk of harmful interventions and enables more informed care strategies that can improve patient outcomes.

In our case, the fact that the diagnosis was made in a dermatology department is also unique, as the specialists most commonly involved are orthopedic surgeons, pediatricians and rheumatologists [7].

To date, there is no specific treatment for FOP. Corticosteroids and selective cyclooxygenase-2 inhibitors are used globally to treat flare-ups and prevent heterotopic ossification due to their anti-inflammatory properties. Preliminary evidence suggests that tofacitinib may be a promising option for mitigating flare-ups in Fibrodysplasia Ossificans Progressiva (FOP). However, its long-term efficacy and safety remain uncertain, emphasizing the need for further research to fully explore the therapeutic potential of JAK-kinase inhibitors in FOP patients. Surgery is contraindicated as it can lead to additional heterotopic ossification. Meanwhile, several clinical trials investigating gene therapies are currently underway [8]. For now, supportive care and preventive measures remain the cornerstone of managing the condition [9].

## Conclusion

Being an extremely rare and incapacitating condition, Münchmeyer’s disease should be evoked in the presence of unexplained nodules or hypertrophy, associated with congenital big toe malformations. This is a concept that dermatologists need to learn about and raise their awareness of, so that they don’t overlook it and lead to a delay in diagnosis, with all that this can entail in terms of complications for our patients. The discovery of the ACVR1 gene offers hope of developing therapies for this hitherto incurable disease [10].
